# HSDL2 Suppresses Epileptic Seizures Through Phosphorylation‐Dependent Modulation of the PSD95‐NMDAR Signaling Axis

**DOI:** 10.1002/cns.70826

**Published:** 2026-03-09

**Authors:** Yan Xia, Wang Jing, Zhang Hui, Xu Demei, Peng Xi, Wang Liang

**Affiliations:** ^1^ Department of Neurology The First Affiliated Hospital of Chongqing Medical University, Chongqing Key Laboratory of Major Neurological and Mental Disorders, Chongqing Key Laboratory of Neurology Chongqing China; ^2^ Department of Neurology The Second Affiliated Hospital of Chongqing Medical University Chongqing China; ^3^ Key Laboratory of Major Brain Disease and Aging Research (Ministry of Education) Chongqing Medical University Chongqing China

**Keywords:** epilepsy, HSDL2, NMDAR, PSD95, TLE

## Abstract

**Background:**

Temporal lobe epilepsy (TLE) is characterized by synaptic dysfunction for which targeted therapies are lacking. Hydroxysteroid dehydrogenase‐like 2 (HSDL2) was previously identified as a potential regulator in TLE, but its precise functional and mechanistic role remained unexplored.

**Methods:**

We compared HSDL2 protein expression in cortical tissues from patients with drug‐resistant TLE and a kainic acid (KA)‐induced mouse model via western blotting. Cellular localization was determined by immunofluorescence co‐staining with neuronal (NeuN and PSD95), astrocytic (GFAP), and microglial (IBA1) markers. Adeno‐associated virus (AAV) vectors were used to overexpress or knock down HSDL2 in the mouse hippocampus, followed by behavioral seizure assessments using pentylenetetrazol (PTZ) and chronic monitoring of spontaneous recurrent seizures (SRS). Underlying mechanisms were investigated through protein–protein interaction, patch‐clamp electrophysiology, and quantitative co‐immunoprecipitation.

**Results:**

HSDL2 was significantly upregulated in both human TLE foci and the KA‐induced epileptic mouse brain. It was localized to both neurons and astrocytes. In vivo, HSDL2 overexpression prolonged the latency to PTZ‐induced seizures and reduced SRS frequency, whereas its knockdown exacerbated seizure severity and duration. Mechanistically, HSDL2 enhanced the membrane localization of postsynaptic density protein 95 (PSD95) and promoted its phosphorylation. This modification disrupted the physical interaction between PSD95 and the N‐methyl‐D‐aspartate receptor (NMDAR) NR2B and NR2A subunits, leading to a reduction in NMDAR‐mediated synaptic currents and neuronal hyperexcitability.

**Conclusions:**

Our findings identify HSDL2 as a novel endogenous antiseizure protein that confers protection in epilepsy by modulating synaptic excitability. Specifically, HSDL2 regulates the PSD95‐NMDAR complex through post‐translational modification of PSD95, thereby inhibiting excessive NMDAR activity. Its therapeutic modulation may offer a strategy for drug development in TLE.

## Graphical abstract

In a kainic acid‐induced mouse model of epilepsy, upregulation of HSDL2 in the hippocampus prolonged seizure latency, increased the membrane expression of PSD95, and enhanced its phosphorylation. Concurrently, HSDL2 attenuated the interaction between PSD95 and the NMDAR subunits GluN2A/GluN2B, thereby suppressing NMDAR overactivation, reducing epileptiform discharges, and exerting a neuroprotective effect in epilepsy.

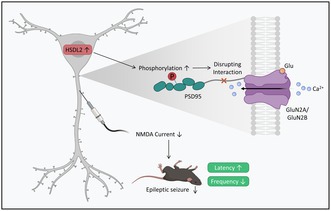

## 1 Introduction

Epilepsy is a highly prevalent and debilitating neurological disorder characterized by abnormally synchronous neuronal discharges in the cerebral cortex [1]. Globally affecting over 70 million individuals, including approximately 10 million in China [2], epilepsy imposes substantial burdens on patients, families, and society. Although more than 30 antiseizure medicines (ASMs) are available, approximately one‐third of patients exhibit pharmacoresistance and ultimately develop drug‐resistant epilepsy [3, 4]. Temporal lobe epilepsy (TLE) represents the most common form of drug‐resistant epilepsy, accounting for 60%–70% of cases, while its precise molecular pathogenesis remains incompletely elucidated [5, 6]. Therefore, elucidating the pathophysiology of TLE and identifying novel therapeutic targets are critical priorities in epilepsy research. Current investigations primarily concentrate on molecular alterations during epileptogenesis [7], potentially neglecting endogenous neuroprotective mechanisms. Emerging evidence indicates that certain molecules demonstrate upregulated expression post‐seizure and may confer neuroprotection, including brain‐derived neurotrophic factor (BDNF), neuropeptide Y (NPY), adenosine, nuclear factor erythroid 2‐related factor 2 (Nrf2), and antioxidant enzymes such as superoxide dismutase (SOD) [8, 9, 10]. Investigating such adaptive responses could reveal novel therapeutic strategies aimed at enhancing the brain's inherent resilience.

Among potential protective candidates, hydroxysteroid dehydrogenase‐like 2 (HSDL2) has recently emerged. Our preliminary tandem mass tag (TMT)‐based proteomic analysis identified significant HSDL2 upregulation in brain tissue from a kainic acid‐induced epilepsy model [11]. This finding aligns with a recent study suggesting a protective role for HSDL2 in epilepsy, potentially mediated through the modulation of astrocytic lipid metabolism [12]. HSDL2 is a mitochondrial and peroxisomal enzyme [13] highly expressed in several tissues, including the brain [14]. Beyond its documented involvement in various cancers [15, 16, 17, 18], HSDL2 appears to have distinct functions within the central nervous system. It has been reported to promote neural repair after spinal cord injury via immunomodulation [19], and genetic studies have linked it to stroke progression and Alzheimer's disease pathology [20, 21]. Despite these associations and its observed upregulation in epilepsy, the direct impact of HSDL2 on neuronal excitability remains entirely unexplored.

To address this critical knowledge gap, we systematically investigated the role of HSDL2 in epilepsy. We first confirmed its consistent upregulation in both human epileptic foci and experimental models. Behavioral assessments across two distinct epilepsy models revealed that HSDL2 overexpression attenuates seizure severity, while its inhibition exacerbates epileptic activity. Electrophysiological analyses demonstrated that HSDL2 primarily modulates N‐methyl‐D‐aspartate receptor (NMDAR)‐mediated currents and functionality. Additionally, we observed HSDL2‐mediated regulation of postsynaptic density protein 95 (PSD95) membrane expression and phosphorylation status. These findings collectively establish HSDL2 as a novel regulator of epileptic seizures and a promising therapeutic target for antiseizure interventions.

## 2 Materials and Methods

### 2.1 Antibodies and Reagents

All antibodies and reagents employed in this study are cataloged in Table S1.

### 2.2 Animal Subjects

Male C57BL/6J wild‐type mice (6–8 weeks old, 20–25 g) were procured from the Experimental Animal Center of Chongqing Medical University. The animals were maintained under specific‐pathogen‐free conditions (5 mice per cage) with controlled environmental parameters: temperature 24°C ± 2°C, relative humidity 60% ± 5%, and a 12‐h light/dark cycle (08:00–20:00 light phase). Food and water were provided adlibitum. Following a minimum acclimatization period of 1 week, all experimental procedures were conducted in strict compliance with protocols approved by the Animal Care and Use Committee of Chongqing University and in accordance with National Institutes of Health guidelines. For tissue collection, mice were deeply anesthetized via intraperitoneal administration of pentobarbital (150 mg/kg) and subsequently euthanized by cervical dislocation. Hippocampal tissues were rapidly excised, immediately placed on ice, and processed for subsequent experimental analyses.

### 2.3 Stereotaxic AAV Administration

Under aseptic surgical conditions, anesthetized mice received bilateral stereotaxic injections of AAV9 vectors (siCtrl, siHSDL2, adCtrl, or adHSDL2) into the hippocampal region. Injection coordinates relative to bregma were as follows: Anteroposterior (AP): −2.0 mm; Mediolateral (ML): ±1.5 mm; Dorsoventral (DV): −1.7 mm (Paxinos Mouse Brain Atlas). A microsyringe delivered 0.5 μL per hemisphere at an infusion rate of 0.2 μL/min, with the needle remaining in situ for 5 min post‐injection to ensure proper viral diffusion. All recombinant AAV vectors were engineered by GeneChem (Shanghai, China). The experimental set comprised (1) overexpression constructs: rAAV‐adHSDL2 and rAAV‐adCtrl; and (2) knockdown constructs: rAAV‐siHSDL2 and rAAV‐siCtrl. The overexpression vectors featured the hSyn promoter‐MCS‐EGFP‐3FLAG‐SV40 PolyA backbone, while knockdown vectors utilized the hSyn promoter‐EGFP‐MIR155(MCS)‐WPRE‐SV40 PolyA architecture. Control viruses contained empty AAV vectors encoding eGFP only.

### 2.4 Kainic Acid Model Establishment

Mice were anesthetized with sodium pentobarbital and immobilized in a digital stereotaxic apparatus (Shenzhen Ruiwode Life Science and Technology Co. Ltd., China) with precise ear bar alignment. Kainic acid (KA; 0.6 μg/μL, Sigma‐Aldrich, USA) was stereotaxically microinjected into the right dorsal hippocampus. Post‐operative animals were maintained on a heating pad at 32°C until full recovery from anesthesia, with continuous monitoring of vital signs. Behavioral seizures were classified according to the modified Racine scale [22, 23]. Three weeks after AAV injection, KA was administered to induce epileptogenesis. Spontaneous recurrent seizures were recorded continuously for 30 days using an infrared video‐EEG monitoring system. Seizure parameters (latency to first seizure, frequency of Racine stage III–V seizures) were analyzed offline by two independent investigators, with one researcher blinded to experimental conditions to ensure objective assessment.

### 2.5 PTZ Model

Three weeks after AAV vector administration, mice were subjected to repeated intraperitoneal (i.p.) injections of pentylenetetrazol (PTZ; 10 mg/kg; Sigma‐Aldrich) at 10‐min intervals. The injection protocol continued until either the onset of generalized seizures or until the cumulative PTZ dose reached 100 mg/kg. Both the latency to first seizure onset and the cumulative PTZ dose required to induce tonic–clonic seizures (Racine stage IV or V) were recorded.

### 2.6 Local Field Potential (LFP) Recording

Surgical procedures were conducted 1 week before data acquisition. Under anesthesia, mice were firmly positioned in a stereotaxic apparatus. Two stainless steel reference electrodes were surgically implanted into the frontal bone. For local field potential (LFP) recordings, an additional electrode was precisely positioned in the right dorsal hippocampal region and connected to a Plexon data acquisition system (Texas, USA). Spontaneous seizure‐like events (SLEs) were identified based on three electrophysiological criteria: (1) amplitude exceeding 200% of baseline activity, (2) frequency surpassing 3 Hz, and (3) duration lasting longer than 5 s.

### 2.7 Western Blotting

The extraction of cell membrane proteins was conducted following the manufacturer's instructions (Proteintech, Wuhan, China). Total protein was extracted using RIPA lysis buffer (Beyotime, Shanghai, China). Protein lysates containing equal quantities were resolved by sodium dodecyl sulfate‐polyacrylamide gel electrophoresis (SDS‐PAGE) and subsequently transferred onto polyvinylidene difluoride (PVDF) membranes (Millipore, USA). To minimize nonspecific binding, membranes were blocked with protein‐free rapid blocking buffer (P0240, Beyotime, Shanghai, China) for 15 min at room temperature. The membranes were then probed with primary antibodies diluted in Beyotime antibody dilution buffer overnight at 4°C. After three 5‐min washes with Tris‐buffered saline containing 0.1% Tween‐20 (TBST), the membranes were incubated with horseradish peroxidase (HRP)‐conjugated secondary antibodies diluted in TBST for 1 h at room temperature. Protein signals were detected using enhanced chemiluminescence reagent (Biosharp, Guangzhou, China) and captured using a Fusion FX7 imaging system (Vilber Lourmat, Marne‐la‐Vallée, France).

### 2.8 Immunostaining

Mice were anesthetized by intraperitoneal injection of 1% pentobarbital sodium. Sequential perfusion was conducted slowly through the left ventricle, first with phosphate‐buffered saline (PBS) and subsequently with 4% paraformaldehyde (PFA). The brains were meticulously extracted, post‐fixed in 4% PFA overnight at 4°C, and cryoprotected in 30% sucrose solution for 48 h. Coronal brain sections were prepared using a Leica cryostat.

For immunohistochemical processing, brain sections were first permeabilized with 0.4% Triton X‐100 for 30 min, followed by antigen retrieval in sodium citrate solution (Boster, Wuhan, China) for 15 min. Nonspecific binding sites were blocked with goat serum working solution (Boster, Wuhan, China) for 60 min at room temperature (RT). Sections were then incubated with primary antibody cocktail diluted in PBS overnight at 4°C. After three 5‐min washes with PBS, sections were incubated with appropriate fluorophore‐conjugated secondary antibodies for 60 min at RT and counterstained with 4′,6‐diamidino‐2‐phenylindole (DAPI).

For immunocytochemical assays, primary neurons were fixed in PBS containing 4% PFA and 4% sucrose for 30 min at RT. Cells were permeabilized with 0.1% Triton X‐100 for 20 min and blocked with goat serum working solution for 60 min at RT. Neurons were incubated with primary antibody cocktail diluted in PBS overnight at 4°C. Following three PBS washes, neurons were incubated with fluorophore‐conjugated secondary antibodies for 60 min at RT. All images were acquired using a Leica confocal microscope (Wetzlar, Germany).

### 2.9 Immunoprecipitation

Hippocampal tissues from C57BL/6 mice were rapidly dissected and homogenized in ice‐cold IP lysis buffer supplemented with complete protease inhibitor cocktail and phosphatase inhibitor cocktail (MedChemExpress). For immunoprecipitation experiments, magnetic protein A/G beads were pre‐incubated with specific primary antibodies (anti‐HSDL2, anti‐PSD95, anti‐NR1, anti‐NR2A, or anti‐NR2B; 3–5 μg each) in PBS buffer at 4°C for 4 h with gentle rotation. After three washes with PBS‐T (PBS containing 0.1% Tween‐20), the antibody‐conjugated beads were incubated with tissue lysates (normalized to 2 mg total protein) overnight at 4°C with constant rotation. Following extensive washing, immunoprecipitated proteins were eluted using 1× Laemmli buffer and analyzed by Western blotting.

For quantitative co‐immunoprecipitation analysis, a standardized protocol was employed where 5 μg of anti‐PSD95 antibody was conjugated to magnetic beads under identical conditions. After removing unbound antibodies, hippocampal lysates (2 mg total protein) were added, and the mixture was incubated for 12 h at 4°C with continuous rotation. The immune complexes were subsequently washed three times with high‐stringency buffer before elution and Western blot analysis.

### 2.10 Mass Spectrometry Analysis

Total protein was extracted from adult wild‐type mouse hippocampal tissues using IP lysis buffer (Beyotime, Shanghai, China). Protein complexes interacting with the anti‐HSDL2 antibody were immunoprecipitated using magnetic bead‐based pull‐down (Dynabeads, Thermo Fisher Scientific). The immunoprecipitated complexes were separated by SDS‐PAGE (4%–20% gradient gel, Bio‐Rad). Following electrophoresis, gels were stained with Coomassie Brilliant Blue R‐250 (Sigma‐Aldrich) at room temperature for 30 min. For mass spectrometry analysis, blank gel regions without visible protein bands were carefully excised to avoid contamination. LC–MS/MS analysis was performed by Applied Protein Technology (Shanghai, China) using a Q Exactive HF‐X mass spectrometer (Thermo Fisher Scientific). The resulting mass spectrometry data were analyzed using Proteome Discoverer 2.4 software, and the MS gene list was subsequently uploaded to the DAVID Bioinformatics Web Server (https://david.ncifcrf.gov/) for comprehensive Gene Ontology (GO) annotation and Kyoto Encyclopedia of Genes and Genomes (KEGG) pathway enrichment analysis.

### 2.11 Quantitative Real‐Time PCR (qPCR)

Total RNA was extracted from hippocampal tissue samples using the RNA Easy Fast Kit (Tiangen, Beijing, China). RNA concentration and purity were quantified using a NanoDrop 2000 spectrophotometer. Subsequently, cDNA synthesis was performed with the FastKing cDNA Kit (Tiangen, Beijing, China) in strict accordance with the manufacturer's protocol. For each reverse transcription reaction, 500 ng of total RNA was converted to cDNA in a 20 μL reaction volume. Quantitative real‐time PCR was conducted using 0.6 μL of cDNA template with SYBR Green FastReal qPCR PreMix (Tiangen, Beijing, China). All samples were analyzed in triplicate, and relative gene expression levels were calculated using the comparative Ct method (2^−ΔΔCt^). For normalization purposes, gene expression data were standardized against the housekeeping gene gapdh. The specific primer sequences used for qPCR analysis are provided in Table S2.

### 2.12 Primary Neuronal Culture

Cortical and hippocampal tissues were aseptically dissected from postnatal day 0 C57BL/6J mouse pups in ice‐cold D‐Hank's Balanced Salt Solution (D‐HBSS). The tissues were mechanically dissociated into 1 mm^3^ fragments and enzymatically digested in 0.25% trypsin solution (Invitrogen; 3 mL/60 mm dish) at 37°C for 10 min with intermittent gentle agitation (every 3 min). Following enzymatic digestion, the cell suspension was plated onto poly‐L‐lysine (Sigma‐Aldrich)‐coated coverslips at a density of 65 cells/mm^2^ in DMEM complete medium supplemented with 20% fetal bovine serum, 1% L‐glutamine, and 1% penicillin–streptomycin. Cells were maintained in a humidified incubator at 37°C with 5% CO_2_. After 4 h of initial plating, the culture medium was replaced with neuronal maintenance medium consisting of Neurobasal Medium supplemented with 2% B‐27, 1% antibiotic‐antimycotic, and 1% L‐glutamine (all from Invitrogen). Semi‐medium changes were performed every 2–3 days by replacing 50% of the conditioned medium with fresh neurobasal medium.

### 2.13 HT22 Cell Culture and Transfection

HT22 cells (obtained from the Chinese Academy of Sciences) were cultured in Dulbecco's modified Eagle's medium supplemented with 10% fetal bovine serum and 1% penicillin–streptomycin and maintained at 37°C in a humidified atmosphere containing 5% CO_2_. For transient transfection experiments, HT22 cells were transfected using Lipofectamine 3000 Reagent (Invitrogen) following the manufacturer's protocol. All plasmids were custom‐designed and constructed by GeneChem (Shanghai, China). The experimental group utilized overexpression vectors with the pAAV‐hSyn promoter‐Hsdl2‐EGFP‐3FLAG‐WPRE‐SV40 PolyA backbone, while the control group received empty AAV vectors encoding eGFP as negative controls.

### 2.14 Whole‐Cell Patch‐Clamp Recordings

Hippocampal slices were prepared from mice that had been transfected with either AAV‐adCtrl or AAV‐adHSDL2 4 weeks prior to experimentation. For whole‐cell patch‐clamp recordings, 300‐μm coronal hippocampal sections were prepared. The slices were then allowed to recover in oxygenated artificial cerebrospinal fluid at 32°C for 30 min, followed by an additional 30 min at room temperature prior to recording. During electrophysiological recordings, the slices were continuously perfused with Mg^2+^‐free aCSF (4 mL/min) at room temperature. To assess NMDAR‐mediated excitatory postsynaptic currents (EPSCs), synaptic responses were evoked using an S48 pulse generator (AstroMed) delivering 400 μs pulses (50–200 μA intensity) at 0.1 Hz through a stimulus isolation unit. Bipolar stimulating electrodes were precisely positioned in the Schaffer collateral pathway. All recordings were performed in the presence of 100 μM picrotoxin (PTX) to block GABAergic inhibition. The NMDAR‐mediated component was isolated by recording at +40 mV in the presence of the AMPAR‐selective antagonist DNQX (20 μM), with the EPSC amplitude measured 50 ms post‐stimulation representing the NMDAR‐mediated current.

### 2.15 Determination of Calcium Concentrations

In HT22 cells, transfection was performed using either the ad‐HSDL2 plasmid or the control plasmid. Following a 48‐h transfection period, the Fluo‐4 AM calcium fluorescent probe solution was prepared in accordance with the manufacturer's protocol from the FLuo‐4 Calcium Detection Kit (Beyotime, Shanghai, China), with all procedures conducted under light‐protected conditions. After washing the cells three times with PBS, they were incubated with 5 μM Fluo‐4 AM (Beyotime, Shanghai, China) at 37°C for 30 min in the dark. Post‐staining, the culture dishes were thoroughly washed and transferred to a microscope chamber containing calcium‐free Hanks' Balanced Salt Solution (Beyotime, Shanghai, China). Real‐time monitoring of intracellular calcium levels was achieved by quantifying Fluo‐4 AM fluorescence intensity using an inverted fluorescence microscope (Leica, Wetzlar, Germany) equipped with a high‐sensitivity sCMOS camera, with images captured at 2‐s intervals for 25 min. L‐Glutamic acid (Sigma, America, 5 mM) was gently added 10 s after the start of filming.

### 2.16 Tandem Mass‐Tag (TMT)‐Based Quantitative Proteomics of Hippocampal Tissue

Hippocampi from WT and KA‐treated mice (*n* = 4 per group) were homogenized in SDT lysis buffer (4% SDS, 100 mM Tris–HCl, 1 mM DTT, pH 7.6). Protein concentration was determined using the BCA assay (Bio‐Rad). Two hundred micrograms of total protein from each sample were processed using filter‐aided sample preparation (FASP; Matthias Mann protocol). Proteins were alkylated with 100 mM iodoacetamide in UA buffer (8 M urea, 150 mM Tris–HCl, pH 8.0), washed, and digested overnight with sequencing‐grade trypsin (1:50, w/w) at 37°C. Peptides were desalted on Empore C18 cartridges and vacuum‐dried.

For quantitative analysis, 100 μg of peptide per sample was labeled with a 6‐plex TMT kit (Thermo Scientific) following the manufacturer's instructions and then pooled. The combined peptides were fractionated using the Thermo High‐pH Reversed‐Phase Peptide Fractionation kit, generating 10 fractions that were further desalted and dried.

Each fraction (1 μg) was analyzed on an Easy‐nLC 1200 coupled to a Q Exactive mass spectrometer (Thermo Scientific). Peptides were trapped on a PepMap 100 C18 column (100 μm × 2 cm) and separated on an Easy‐Spray C18 analytical column (75 μm × 10 cm, 3 μm) using a 60 min linear gradient of 5%–38% solvent B (0.1% formic acid in 84% acetonitrile) at 300 nL/min. The Q Exactive operated in positive ion, data‐dependent top‐10 mode with the following settings: survey scan m/z 300–1800 at 70 k resolution (m/z 200); AGC target 3 × 10^6^; maximum IT 10 ms; HCD MS/MS resolution 17,500; isolation window 2.0 m/z; NCE 30 eV; dynamic exclusion 40 s.

Raw files were searched in Proteome Discoverer 1.4 with Mascot 2.2 against the UniProt mouse reference proteome. Search parameters: fixed modifications—Carbamidomethyl (C), TMT‐6plex (N‐term/K); variable modifications—Oxidation (M), TMT‐6plex (Y); enzyme—trypsin, ≤ 2 missed cleavages; precursor tolerance ±20 ppm; fragment tolerance 0.1 Da. Peptide‐ and protein‐level FDRs were set to ≤ 1% using a decoy strategy. Protein quantification used only unique peptides, and reporter ion intensities were normalized to the median protein ratio. Differentially expressed proteins were defined by |log_2_ FC| ≥ 1 and adjusted *p* < 0.05.

### 2.17 Statistics

Statistical analyses were conducted using GraphPad Prism 9.0 (GraphPad Software Inc., United States). All quantitative data are expressed as mean ± standard deviation (SD). Intergroup comparisons were performed using Student's *t*‐test for two‐group analyses, while one‐way analysis of variance (ANOVA) was employed for multiple group comparisons. A *p* value < 0.05 was considered statistically significant.

## 3 Results

### 3.1 Expression of HSDL2 in Brain Tissues of TLE Patients and Epileptic Mice

**FIGURE 1:**
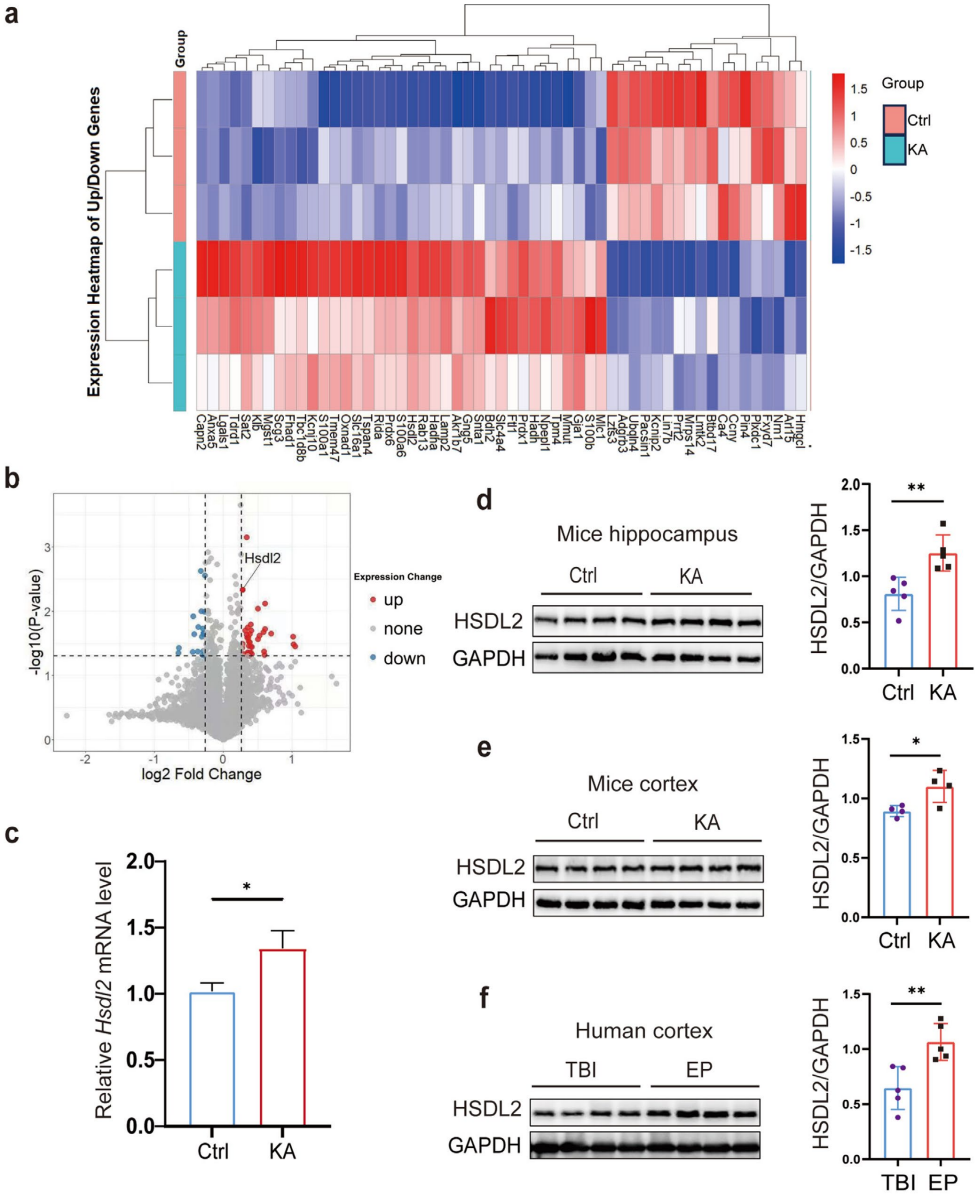
HSDL2 expression is significantly upregulated in the brain tissues of KA‐induced epileptic mice and patients with TLE. (a) Heat map visualization of 78 significantly differential proteins from the quantitative proteomic analysis using TMT labeling. (b) Quantitative proteomic analysis using TMT labeling demonstrated a significant upregulation of HSDL2 protein expression in the hippocampus of KA‐treated epileptic mice compared to control mice. (c) Quantitative real‐time PCR analysis revealed elevated *Hsdl2* mRNA levels in hippocampal tissues of KA model mice (*n* = 5/group, *p < 0.05, unpaired two‐tailed Student's *t*‐test). (d) Representative immunoblot showing HSDL2 protein expression in mouse hippocampal tissues and quantitative analysis (*n* = 5/group, **p < 0.01, unpaired two‐tailed Student's *t*‐test). (e) Representative immunoblot of HSDL2 expression in mouse cortical tissues and quantitative analysis (*n* = 5/group, *p < 0.05, unpaired two‐tailed Student's *t*‐test). (f) Representative immunoblot analysis of HSDL2 expression in human temporal lobe tissues and quantitative analysis (*n* = 5/group, **p < 0.01, unpaired two‐tailed Student's *t*‐test).

Initial TMT‐based differential proteomic analysis demonstrated significant upregulation of HSDL2 protein levels in the hippocampus of KA model mice (Figure 1a,b). To systematically validate HSDL2 expression patterns in brain tissue, we conducted comprehensive transcriptional and protein‐level analyses using qPCR and Western blotting on hippocampal tissues from both KA‐treated and control mice. Our results revealed a statistically significant increase in HSDL2 transcript levels in the hippocampus of the KA model group compared to controls (Figure 1b). Correspondingly, Western blot analysis confirmed elevated HSDL2 protein expression in the KA‐treated group (Figure 1c,d). Furthermore, we extended our investigation to cortical tissues, where immunohistochemical analysis consistently showed upregulated HSDL2 protein expression in both KA‐treated mice and TLE patients (Figure 1e,f, Table S3).

### 3.2 Subcellular Localization of HSDL2 in Brain Tissues and Primary Neurons of Wild‐Type Mice

**FIGURE 2:**
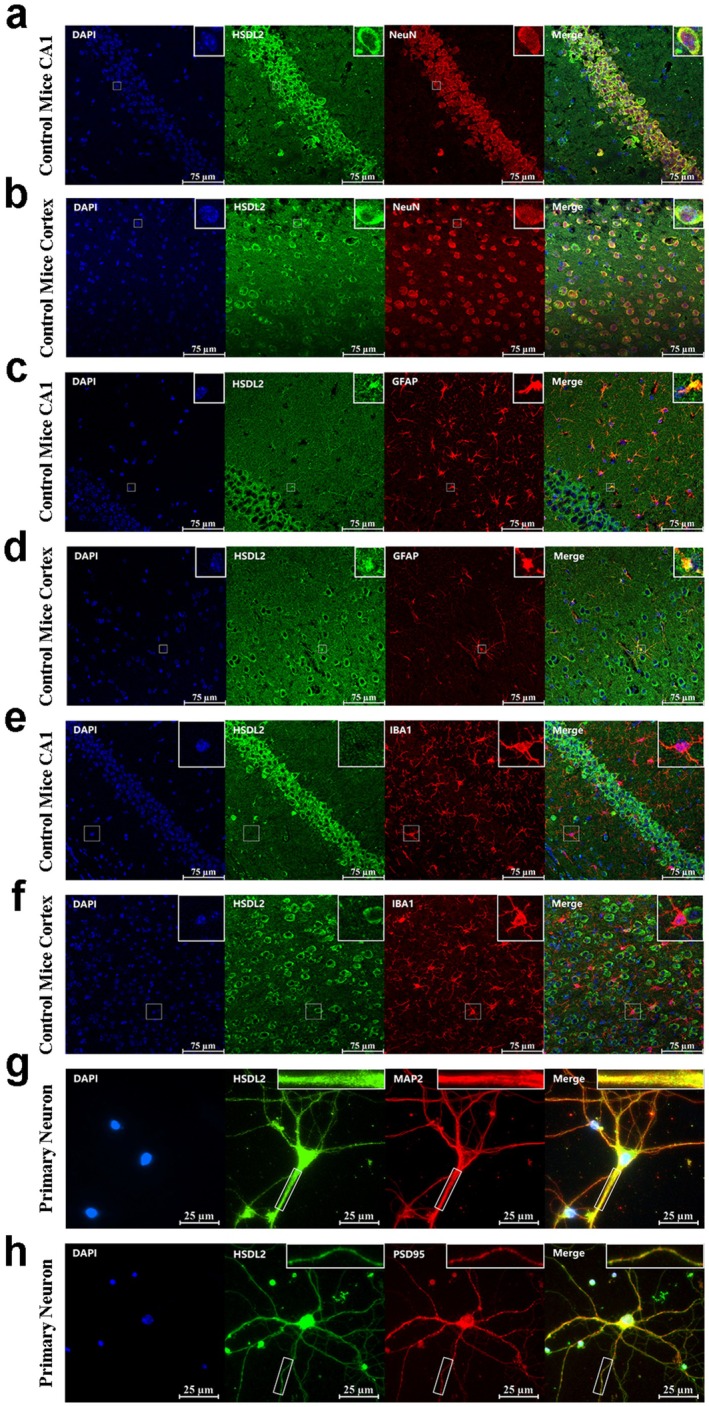
Distribution and subcellular localization of HSDL2 in mouse brain tissues. (a, b) Representative immunofluorescence images of HSDL2, DAPI, and NeuN in the hippocampus and cortex of control mice. (c, d) Representative immunofluorescence images of HSDL2, DAPI, and GFAP in the hippocampus and cortex of control mice. (e, f) Representative immunofluorescence images of HSDL2, DAPI, and IBA1 in the hippocampus and cortex of control mice. (g) Representative immunofluorescence images of HSDL2, DAPI, and MAP2 in primary neurons. (h) Representative immunofluorescence images of HSDL2, DAPI, and PSD95 in primary neurons.

In wild‐type mouse brain sections, HSDL2 immunoreactivity was ubiquitously detected across various hippocampal subregions and cortical areas (Figure S1). Detailed co‐localization studies demonstrated that HSDL2 exhibited significant spatial overlap with the neuronal marker NeuN (Figure 2a,b) and the astrocytic marker GFAP (Figure 2c,d), while showing negligible co‐localization with the microglial marker IBA1 (Figure 2e,f). Notably, the HSDL2 expression profile in brain sections from KA‐induced epileptic mice mirrored the distribution pattern observed in wild‐type controls (Figure S2). Quantitative fluorescence intensity measurements revealed a statistically significant elevation of HSDL2 signal intensity in both hippocampal and cortical neurons of KA‐treated mice compared to wild‐type mice. In contrast, astrocytic HSDL2 expression levels remained comparable between experimental groups (Figure S3).

To further characterize the neuronal compartmentalization of HSDL2, we performed dual‐labeling experiments in primary neuronal cultures using the dendritic/axonal marker MAP2. This analysis confirmed the co‐localization of HSDL2 with MAP2‐positive neuronal processes (Figure 2g). Additionally, synaptic localization studies identified significant co‐distribution of HSDL2 with the excitatory postsynaptic density marker PSD95 (Figure 2h).

### 3.3 HSDL2 Modulates Susceptibility and Severity of Epileptic Seizures

**FIGURE 3:**
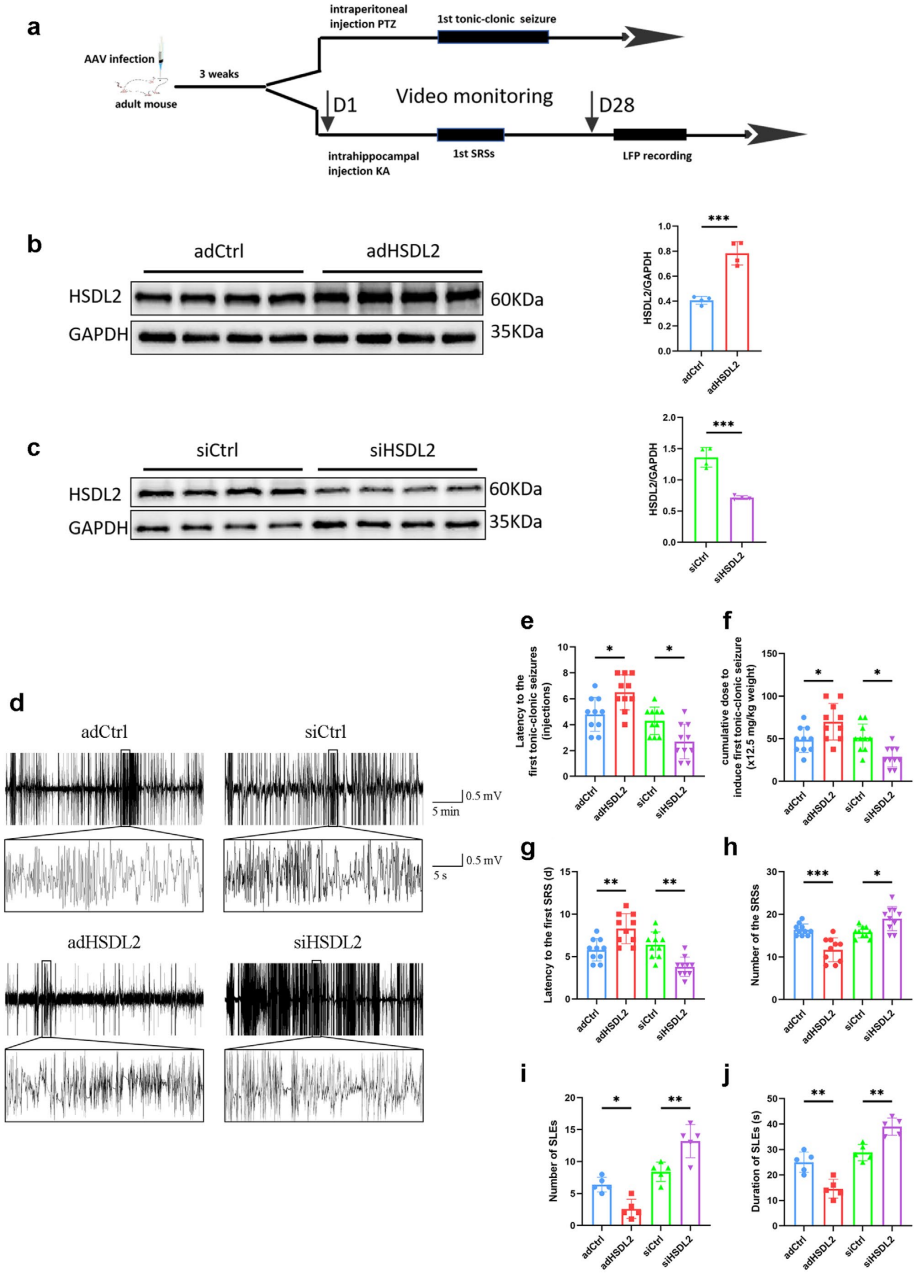
HSDL2 modulates seizure susceptibility and severity. (a) Schematic diagram illustrating the experimental timelines for the two distinct mouse models employed in this study. (b) Representative immunoblot images demonstrating HSDL2 protein expression profiles in the hippocampus of AAV‐adHSDL2 transduced mice and quantitative analysis (*n* = 6/group, ***p < 0.001, unpaired two‐tailed Student's *t*‐test). (c) Representative immunoblot images demonstrating HSDL2 protein expression profiles in the hippocampus of AAV‐siHSDL2 transduced mice and quantitative analysis (*n* = 5/group, ***p < 0.001, unpaired two‐tailed Student's *t*‐test). (d) Representative local field potential (LFP) recordings depicting spontaneous recurrent seizures in KA‐model mice following AAV‐siHSDL2 or AAV‐adHSDL2 transduction. (e) The impact of HSDL2 modulation on seizure thresholds in the PTZ model (*n* = 10/group, *p < 0.05, unpaired two‐tailed Student's *t*‐test). (f) Cumulative PTZ dosage required to elicit tonic–clonic seizures following HSDL2 modulation (*n* = 10/group, *p < 0.05, unpaired two‐tailed Student's *t*‐test). (g) Latency to spontaneous recurrent seizures (SRS) and (h) daily SRS frequency monitored over a 30‐day period following status epilepticus (*n* = 10/group, *p < 0.05, **p < 0.01, ***p < 0.001, unpaired two‐tailed Student's *t*‐test). (i) Quantitative assessment of both the number and (j) duration of seizure‐like events (SLEs) across experimental groups (*n* = 5/group, *p < 0.05, **p < 0.01, unpaired two‐tailed Student's *t*‐test).

To systematically investigate the role of HSDL2 in seizure pathogenesis, we employed stereotaxic delivery of AAV vectors to either overexpress or knock down HSDL2 in the mouse hippocampus. Seizure susceptibility was quantitatively assessed using two well‐established models: the KA and PTZ models (Figure 3a). Three weeks post‐injection, robust GFP expression confirmed successful and widespread transduction throughout hippocampal subregions (Figure S4). Quantitative immunoblot analysis revealed a significant increase in hippocampal HSDL2 protein levels following overexpression and a significant decrease after knockdown compared to control groups (Figure 3b,c).

Behavioral analysis demonstrated that HSDL2‐overexpressing mice exhibited significantly prolonged latency to first clonic seizure onset and required higher cumulative PTZ doses to induce tonic–clonic seizures compared to control mice. Conversely, HSDL2‐knockdown mice displayed reduced latency to the first clonic seizure and required lower cumulative PTZ doses to elicit tonic–clonic seizures (Figure 3e,f).

We further evaluated the modulation of SRS through comprehensive behavioral monitoring and LFP recordings. Quantitative analysis showed that HSDL2‐overexpressing mice displayed prolonged SRS latency and reduced SRS frequency compared to controls. In contrast, HSDL2‐knockdown mice exhibited significantly shortened SRS latency and increased SRS frequency (Figure 3g,h). LFP spectral analysis (Figure 3d) provided electrophysiological confirmation of these behavioral observations: the overexpression group demonstrated fewer SLEs and prolonged SLE latency, while the knockdown group showed increased SLE frequency and reduced SLE latency relative to controls (Figure 3i,j).

### 3.4 HSDL2 Does Not Regulate the Protein Expression of the Glutamatergic NMDA Receptor Subunits NR2A, NR2B, and NR1

**FIGURE 4:**
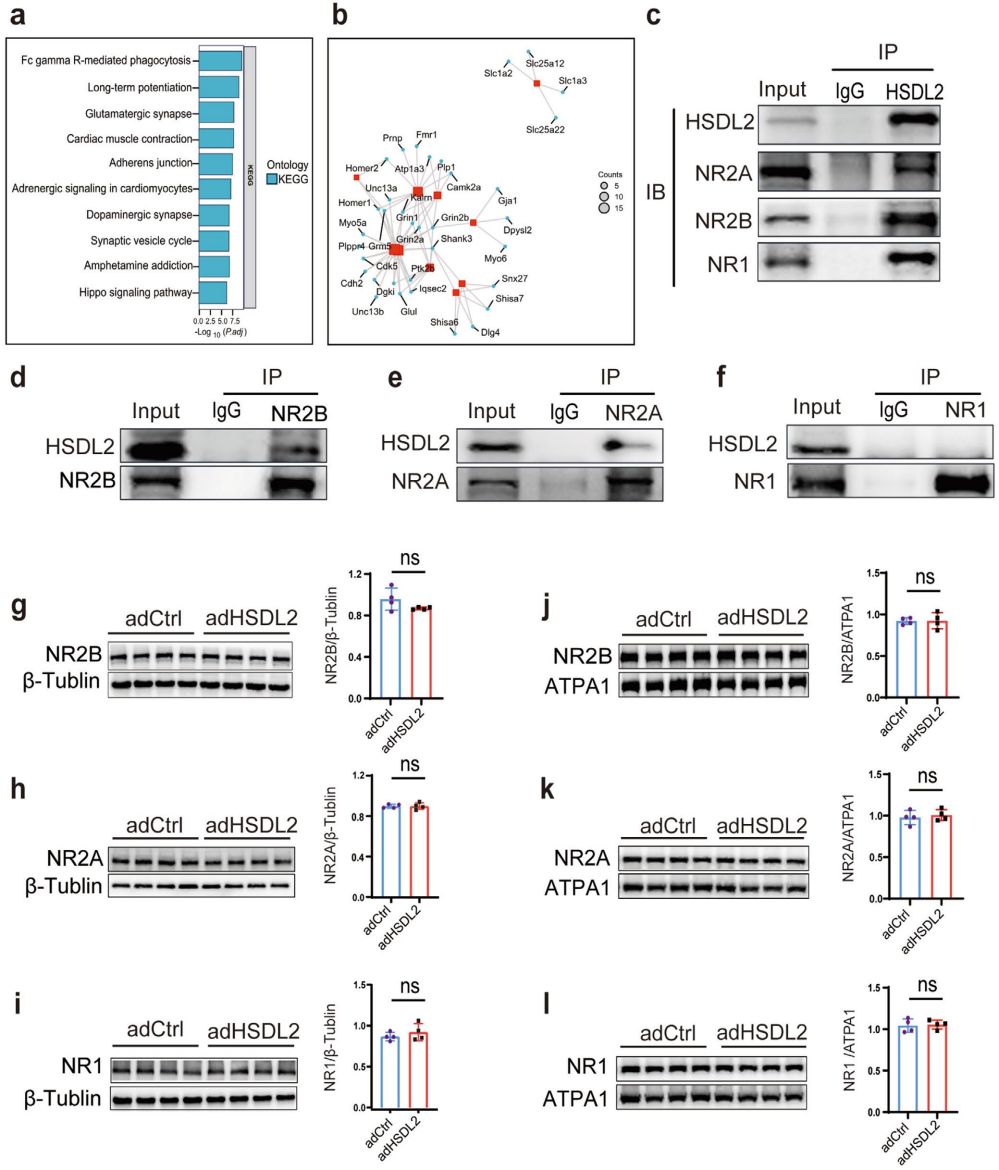
HSDL2 interacts with NMDA receptor subunits without modulating their expression levels. (a) KEGG pathway analysis of putative HSDL2‐interacting proteins identified through ESI‐MS in mouse hippocampal tissue. (b) Protein–protein interaction (PPI) network analysis within the glutamatergic synapse pathway, as revealed by KEGG enrichment. (c) Characterization of interactions between HSDL2 and the indicated proteins by Co‐IP. Hippocampal lysates from wild‐type mice were precipitated with anti‐HSDL2 magnetic beads, and precipitates were subsequently immunoblotted with antibodies to detect NR2A, NR2B, and NR1. IgG immunoprecipitate was used as a negative control. (d–f) Reverse validation of the interaction of NR2B, NR2A, and NR1 with HSDL2 in lysates from the hippocampal lysates from wild‐type mice by Co‐IP assay. (g–i) Representative Western blot images and statistical graphs of total NMDA receptor subunits (NR2B, NR2A, and NR1) protein expression in the rAAV‐adHSDL2 group and corresponding empty virus group. (*n* = 4/group, ns *p* > 0.05). (j–l) Representative Western blot images and statistical graphs of membrane NMDA receptor subunits (NR2B, NR2A, and NR1) protein expression in the rAAV‐adHSDL2 group and corresponding empty virus group. (*n* = 5/group, ns *p* > 0.05).

To identify potential HSDL2‐interacting proteins, we conducted co‐immunoprecipitation (Co‐IP) combined with electrospray ionization mass spectrometry (ESI‐MS) using hippocampal lysates from wild‐type mice. This proteomic analysis identified 860 candidate interacting proteins (Table S4). Subsequent Kyoto Encyclopedia of Genes and Genomes (KEGG) pathway enrichment analysis demonstrated significant enrichment of these interactors in glutamatergic synapse and synaptic vesicle cycling pathways (Figure 4a, Table S5). Of particular interest, the glutamatergic synapse pathway included NMDA receptor subunits NR1, NR2A, and NR2B, along with the scaffolding protein PSD95 (Figure 4b).

Further immunoprecipitation experiments confirmed that HSDL2 could specifically precipitate NR1, NR2A, and NR2B when used as bait (Figure 4c). Reciprocal Co‐IP experiments, using either NR2A or NR2B as bait, successfully precipitated HSDL2 (Figure 4d,e). However, when NR1 was employed as bait, HSDL2 precipitation was not observed (Figure 4f).

To examine whether HSDL2 overexpression modulates NMDA receptor subunits, we performed western blot analysis. The results indicated that HSDL2 overexpression neither affected the total protein levels of NR1, NR2A, and NR2B (Figure 4g–i) nor altered their membrane expression (Figure 4j–l).

### 3.5 HSDL2 Modulates NMDA Receptor Current Amplitude and Function

**FIGURE 5:**
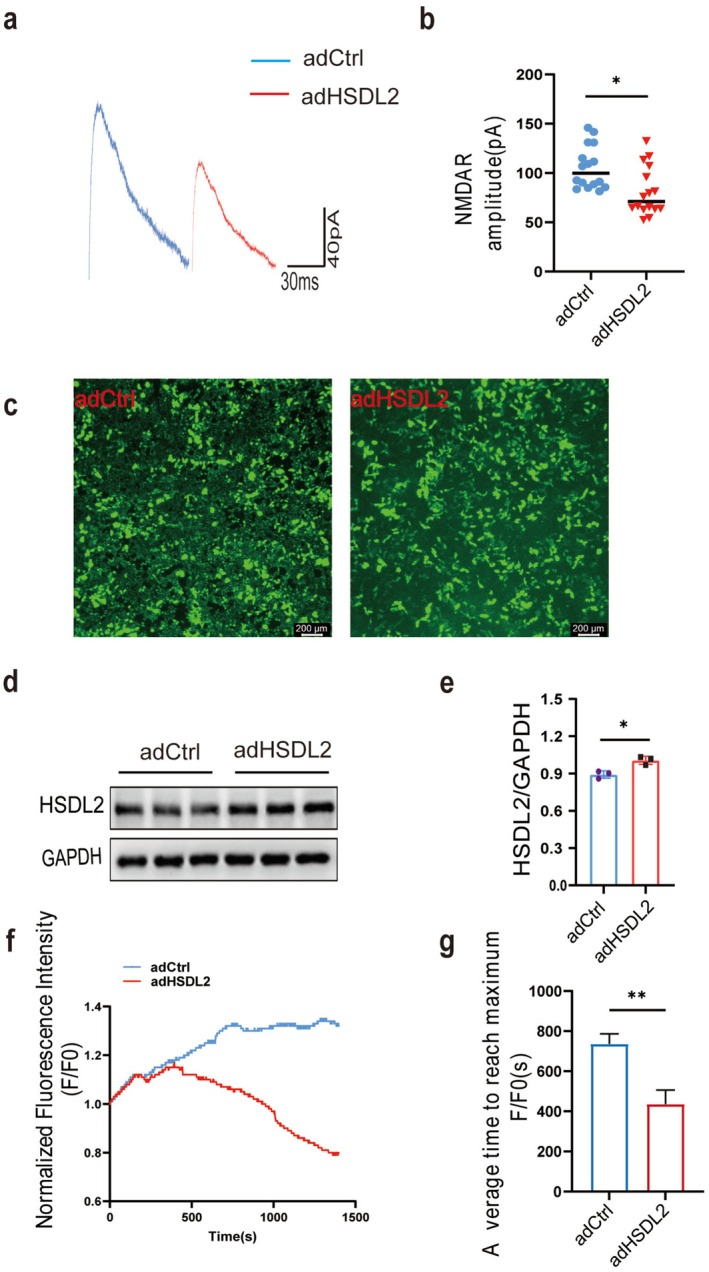
HSDL2 overexpression attenuates NMDA receptor‐mediated postsynaptic currents and calcium influx in HT22 hippocampal neuronal cells. (a) Whole‐cell patch‐clamp electrophysiological recordings of NMDA receptor‐evoked excitatory postsynaptic currents (EPSCs) in control and rAAV‐adHSDL2 groups. (b) Statistical comparison of mean NMDA current amplitudes (*n* = 16 cells, 4 mice/group, **p < 0.05, unpaired two‐tailed Student's *t*‐test). (c) Representative immunofluorescence images demonstrating successful transfection of HT22 cells with HSDL2‐overexpression plasmid compared to empty vector control. Scale bar = 200 μm. (d) Western blot analysis confirming significant HSDL2 protein overexpression. (e) Densitometric quantification of HSDL2 protein expression levels (*n* = 3, *p < 0.05, unpaired two‐tailed Student's *t*‐test). (f) Quantitative assessment of L‐Glutamic acid (5 mM) intracellular calcium transients measured by Fluo‐4 AM fluorescence. Data are presented as normalized calcium flux (F/F₀ ratio) in adHSDL2 versus control groups (*n* = 3 times). (g) The bar plot shows the average time to reach maximum F/F_0_ (*n* = 3, **p < 0.01, unpaired two‐tailed Student's *t*‐test).

Whole‐cell patch‐clamp recordings performed in mouse hippocampal CA1 neurons demonstrated that HSDL2 overexpression significantly attenuated NMDA‐evoked current amplitude (Figure 5a,b). Consistent with these electrophysiological observations, calcium imaging experiments in HT22 cells transfected with HSDL2‐expression plasmids revealed a pronounced reduction in intracellular calcium flux upon glutamate stimulation at equivalent concentrations compared to control groups (Figure 5c–g). These complementary experimental approaches provide converging evidence that HSDL2 overexpression suppresses both NMDA receptor‐mediated currents and subsequent neuronal calcium influx. Notably, these functional modifications were observed in the absence of any detectable alterations in NMDA receptor subunit protein expression levels.

### 3.6 HSDL2 Negatively Modulates Glutamatergic NMDAR Synaptic Transmission by Enhancing Membrane Expression and Phosphorylation of PSD95

**FIGURE 6:**
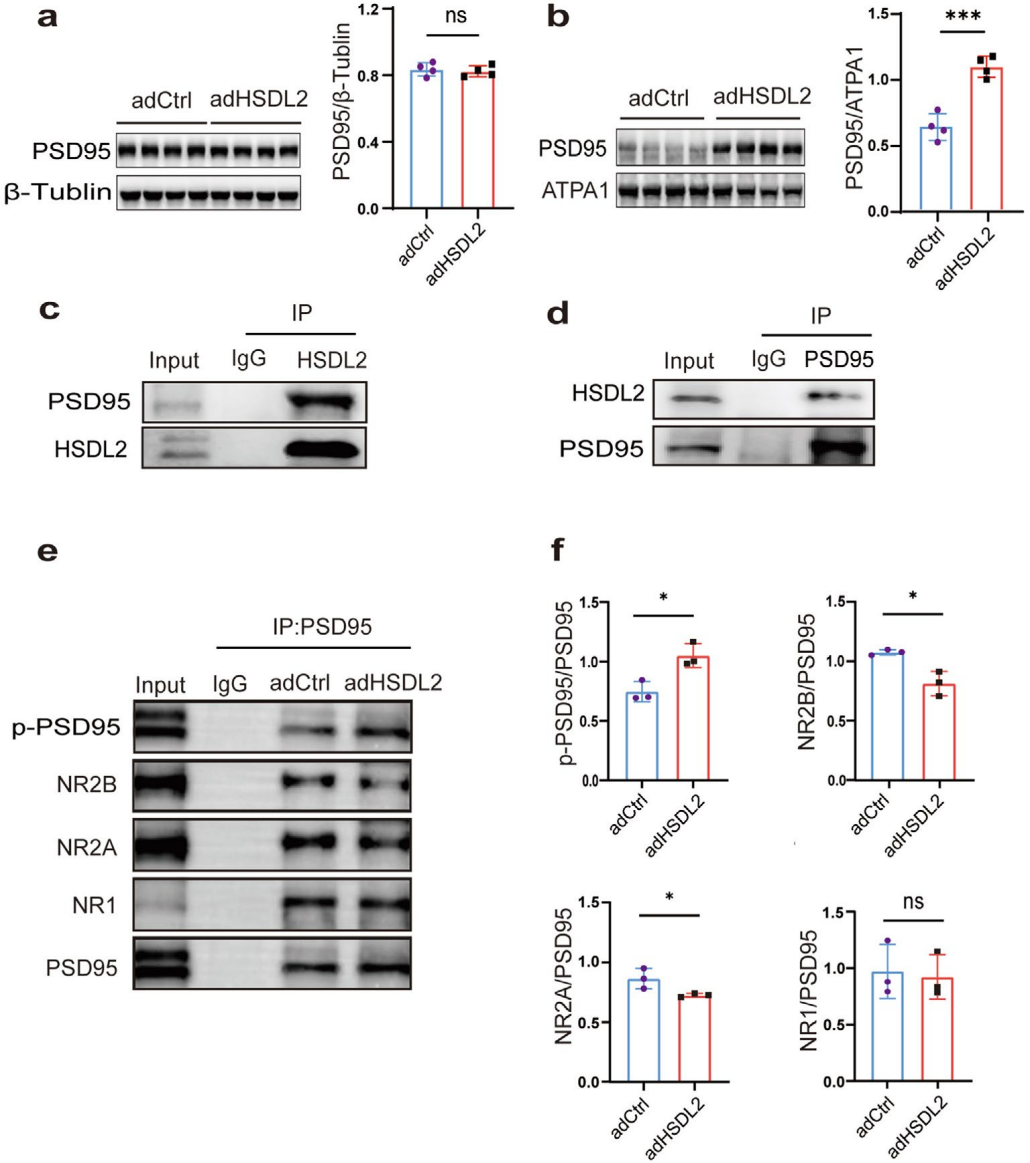
HSDL2 overexpression enhances PSD95 membrane localization and phosphorylation to negatively regulate PSD95‐NR2A/B complexes. (a) Western blot analysis and quantitative assessment demonstrated comparable total PSD95 protein levels between rAAV‐adHSDL2 and empty vector control groups (*n* = 4, ns *p* > 0.05, unpaired two‐tailed Student's *t*‐test). (b) Western blot analysis and quantitative assessment demonstrated comparable membrane PSD95 protein levels between rAAV‐adHSDL2 and empty vector control groups (*n* = 5, ***p < 0.001, unpaired two‐tailed Student's *t*‐test). (c, d) Co‐IP was used to verify the interactions of HSDL2 with PSD95. Hippocampal lysates from WT mice were precipitated using anti‐HSDL2‐bound magnetic beads, and subsequent immunoblotting with antibodies against PSD95 was performed. IgG immunoprecipitate was used as a negative control. (e) Quantitative Co‐IP experiments demonstrated that HSDL2 overexpression significantly enhanced PSD95 phosphorylation while reducing its binding affinity to NR2A and NR2B subunits, without affecting NR1 interaction. Hippocampal lysates from rAAV‐adHSDL2‐ and rAAV‐adCtrl‐treated mice were precipitated with anti‐PSD95 magnetic beads, followed by immunoblotting using antibodies against phospho‐(Ser/Thr) Phe, NR2B, NR2A, NR1, and PSD95. IgG immunoprecipitate was used as a negative control. (f) Representative Western blot statistical graphs of p‐PSD95, NR2B, NR2A, and NR1 protein expression in the rAAV‐adHSDL2 group and corresponding empty virus group. (*n* = 3, ns *p* > 0.05, *p < 0.05, unpaired two‐tailed Student's *t*‐test)

The scaffolding protein PSD95 plays an essential role in anchoring NMDA receptors to the postsynaptic membrane and maintaining their functional integrity. Our experiments revealed that HSDL2 overexpression specifically elevated membrane‐associated PSD95 protein levels in hippocampal neurons (Figure 6b), while total PSD95 expression remained unchanged (Figure 6a). Co‐immunoprecipitation assays confirmed a direct physical interaction between HSDL2 and PSD95 (Figure 6c,d), which was further supported by their endogenous co‐localization in primary neuronal cultures (Figure 2h). Importantly, quantitative co‐IP analysis using PSD95 as bait demonstrated three key findings: (1) HSDL2 overexpression significantly increased PSD95 phosphorylation (Figure 6e,f), (2) reduced PSD95 binding affinity for NR2A/NR2B subunits (Figure 6e,f), while (3) maintaining normal interaction with NR1 subunits (Figure 6e,f).

## 4 Discussion

Our study identifies HSDL2 as an endogenous neuroprotective factor that confers antiseizure properties through neuronal‐specific mechanisms. The primary mechanistic pathway involves attenuation of epileptogenic susceptibility via modulation of PSD95‐NMDAR complex functionality, thereby suppressing pathological NMDAR overactivation. This discovery not only elucidates a previously unrecognized neuroprotective role for HSDL2 in epilepsy pathogenesis but also establishes a molecular framework for developing synapse‐targeted antiepileptic therapies.

This study systematically investigated the expression patterns and functional roles of HSDL2 in TLE. Immunohistochemical analyses revealed significant upregulation of HSDL2 in the cortex of TLE patients and in both hippocampal and cortical regions of KA‐induced epileptic mice. Through comprehensive immunofluorescence co‐localization studies, we established that HSDL2 exhibits predominant neuronal localization, with particularly strong co‐expression observed with the glutamatergic synaptic marker PSD95. While detectable in astrocytes, the neuronal enrichment of HSDL2 was substantially more pronounced. Importantly, microglial cells showed negligible HSDL2 expression. Functional assessments demonstrated that HSDL2 knockdown significantly aggravated seizure susceptibility, whereas its overexpression exerted robust neuroprotective effects against epileptogenesis. These findings collectively suggest that HSDL2 serves as a bona fide endogenous antiseizure factor, rather than constituting a secondary disease correlate. Notably, the observed neuroprotective mechanism appears distinct from previously reported HSDL2‐mediated regulation of astrocytic lipid metabolism [12], highlighting the pivotal role of direct neuronal modulation in its antiseizure action. To elucidate the molecular mechanism underlying HSDL2's function, we conducted comprehensive Co‐IP/MS screening. This systematic approach identified robust interactions between HSDL2 and key components of the glutamatergic synaptic pathway. Specifically, HSDL2 exhibited significant binding affinity with NMDA receptor subunits (NR1, NR2A, and NR2B), while showing no detectable interaction with AMPA receptor subunits (Figure S5). Intriguingly, despite these interactions, HSDL2 overexpression failed to modulate either the total expression levels or membrane localization of NMDAR subunits, indicating that its regulatory mechanism operates independently of NMDAR protein abundance. To further investigate this phenomenon, we performed whole‐cell patch‐clamp electrophysiological analysis in vitro, which revealed that HSDL2 overexpression significantly attenuated the amplitude of NMDA‐mediated postsynaptic currents. Glutamatergic NMDAR‐mediated Ca^2+^ influx plays a pivotal role in neuronal function [24, 25]. To investigate whether elevated HSDL2 expression influences NMDAR functionality, we employed the Fluo‐4 AM calcium indicator to monitor intracellular calcium dynamics. Our results demonstrated that HSDL2 overexpression significantly attenuated the amplitude of intracellular calcium transients relative to control conditions. These findings suggest that upregulation of HSDL2 expression compromises NMDAR‐mediated calcium signaling pathways. HSDL2 negatively regulates NMDAR function through a mechanism independent of modulating receptor subunit abundance or membrane localization. To elucidate this mechanism, we must consider the critical role of PSD95, a postsynaptic scaffolding protein that directly interacts with the carboxyl termini of NMDA receptor subunits NR2A and NR2B via its PDZ domains. This molecular interaction serves to anchor NMDARs at specific postsynaptic membrane sites, thereby maintaining their synaptic concentration and preserving their functional integrity [26, 27]. The stability of the NMDAR‐PSD95 complex exhibits a well‐documented association with epileptogenesis. Existing evidence has demonstrated a significant reduction in the expression level of PSD95 within the hippocampal region of kainic acid‐induced epileptic model mice [28, 29]. Furthermore, accumulating research highlights the pivotal involvement of NR2 subunits in epilepsy pathogenesis [30], with particular emphasis on how modifications in NMDA subunit‐PSD95 binding affinity modulate seizure susceptibility. Experimental studies have established that strengthened NR2A‐PSD95 interactions exacerbate spontaneous recurrent seizures in epileptic animal models [31], while augmented NR2B‐PSD95 binding similarly potentiates spontaneous seizure activity during chronic epileptic phases [32].

Does HSDL2 regulate the PSD95‐NMDAR interaction? Our investigation demonstrated that although HSDL2 overexpression had no significant effect on NMDAR subunit expression levels, it markedly enhanced PSD95 membrane localization. Notably, PSD95 is predominantly localized at excitatory postsynaptic densities in dendritic spines [33]. Through quantitative Co‐IP analysis, we further observed that HSDL2 overexpression significantly attenuated the binding affinity between PSD95 and NMDAR subunits NR2A/NR2B, while maintaining normal interaction with the NR1 subunit. These findings collectively indicate that HSDL2 expression promotes PSD95 membrane accumulation while simultaneously impairing its functional coupling with NMDAR complexes. We subsequently examined the potential regulatory role of HSDL2 in post‐translational modifications of PSD95. As a scaffold protein containing multiple phosphorylation sites, PSD95 undergoes functional modulation through dynamic phosphorylation changes [34]. Notably, dephosphorylation at the Tyr533 residue has been demonstrated to impair PSD95 synaptic targeting and reduce synaptic density [35]. Furthermore, site‐specific phosphorylation at serine/threonine residues orchestrates the dynamic regulation of PSD95/NMDAR synaptic clustering. This phosphorylation‐dependent mechanism critically modulates the PSD95‐NMDAR interaction, serving as a pivotal regulator of excitatory synaptic transmission and plasticity [36, 37, 38, 39].

This study has several notable limitations: First, the specific phosphorylation site(s) on PSD95 modulated by HSDL2 were not identified. Second, potential contributions from other types of PSD95 post‐translational modifications, including palmitoylation and ubiquitination, were not systematically excluded. Third, the dynamic functional role of HSDL2 across distinct temporal phases of epilepsy (acute versus chronic) remains unexplored. Furthermore, the development of blood–brain barrier (BBB)‐penetrating HSDL2 agonists presents a significant translational challenge for future clinical applications. In summary, this study presents novel evidence demonstrating that HSDL2 upregulation operates through a dual regulatory mechanism: it simultaneously enhances PSD95 membrane expression while inhibiting its binding to NR2A/2B via phosphorylation. This coordinated bidirectional modulation serves to maintain synaptic structural integrity while suppressing neuronal hyperexcitability, thereby elucidating the molecular basis for HSDL2's dual neuroprotective effects.

## Author Contributions

W.L. and P.X. designed this project; Y.X. and W.J. wrote the manuscript; W.L. and X.D.M. revised the manuscript; W.J. and Z.H. performed the animal experiments; Y.X. and W.J. performed the molecular biology experiments; W.L. and X.D.M. performed the patch clamp; and Y.X. and W.J. analyzed the data and performed the statistical analyses. All the authors have read and approved the final manuscript.

## Funding

This work was supported by the High‐Level Medical Reserved Personnel Training Project of Chongqing, Chongqing Municipal Health Commission (2020GDRC019) and the Chongqing Postdoctoral Program Funding, Chongqing Municipal Human Resources and Social Security Bureau (2010010005343386).

## Ethics Statement

All the animal experiments were approved by the Ethics Committee of Chongqing Medical University (Approval No.: IACUC‐CQMU‐2023‐0146). Human brain tissue samples from patients with TLE or TBI were collected from the First Affiliated Hospital of Chongqing Medical University. Patients provided written informed consent for the utilization of brain tissue and access to medical records for research objectives. The collection and use of all the samples were approved by the Ethics Committee of the First Affiliated Hospital of Chongqing Medical University (Approval No.: 2024‐271‐01) and were performed in accordance with the Declaration of Helsinki.

## Conflicts of Interest

The authors declare no conflicts of interest.

## Supporting information


**Figure S1:** Distribution of HSDL2 in control mouse brain tissues' immunofluorescence. HSDL2 (green), DAPI (blue).
**Figure S2:** Distribution and localization of HSDL2 in KA mice brain tissues.
**Figure S3:** Quantitative Fluorescence Analysis of HSDL2 in Brain Sections of KA Mice and control Mice.
**Fig**ure **S4**. Immunofluorescence staining of enhanced green fluorescent protein (eGFP) in the hippocampus following transfection with AAV‐eGFP‐adHSDL2 (overexpression) or AAV‐eGFP‐siHSDL2 (knockdown).
**Figure S5:** Co‐immunoprecipitation (Co‐IP) assays confirming physical interactions between HSDL2 and GluA1and GluA2 subunits.
**Table S1:** Antibody details.
**Table S2:** Sequences of qPCR primers.
**Table S3:** Clinical characteristics of TLE patients and TBI patients.

## Data Availability

The data that support the findings of this study are available from the corresponding author upon reasonable request.
