# Supplementary material for: HSDL2 Suppresses Epileptic Seizures Through Phosphorylation‐Dependent Modulation of the PSD95‐NMDAR Signaling Axis
Source: CNS Neurosci Ther. 2026 Mar 9;32(3):e70826. doi: 10.1002/cns.70826 (PMC12971609; doi:10.1002/cns.70826)
Supplement: Supplementary file 1 — Figure S1: Distribution of HSDL2 in control mouse brain tissues' immunofluorescence. HSDL2 (green), DAPI (blue). Figure S2: Distribution and localization of HSDL2 in KA mice brain tissues. Figure S3: Quantitative Fluorescence Analysis of HSDL2 in Brain Sections of KA Mice and control Mice. Figure S4. Immunofluorescence staining of enhanced green fluorescent protein (eGFP) in the hippocampus following transfection with AAV‐eGFP‐adHSDL2 (overexpression) or AAV‐eGFP‐siHSDL2 (knockdown). Figure S5: Co‐immunoprecipitation (Co‐IP) assays confirming physical interactions between HSDL2 and GluA1and GluA2 subunits. Table S1: Antibody details. Table S2: Sequences of qPCR primers. Table S3: Clinical characteristics of TLE patients and TBI patients. [file CNS-32-e70826-s001.zip › cns70826-sup-0001-FigureS1-S5-TableS1-S3@Supplementary information.docx]

# **Supplemental information**

## **Supplemental figures**


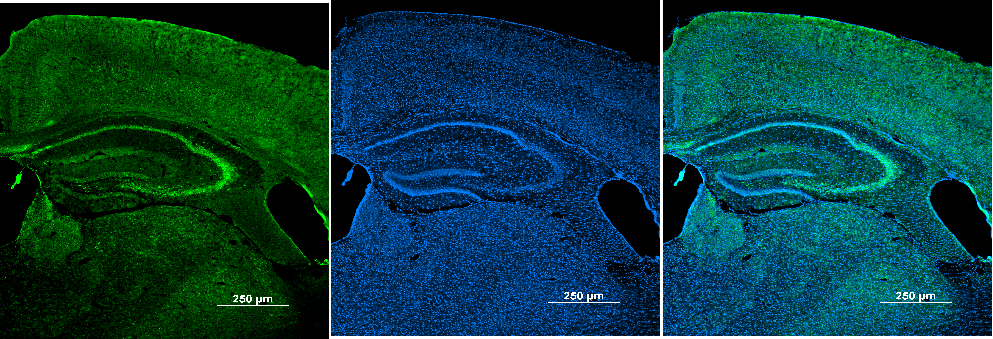


**Fig.S1: Distribution of HSDL2 in control mouse brain tissues´****immunofluorescence. HSDL2 (green), DAPI (blue).**


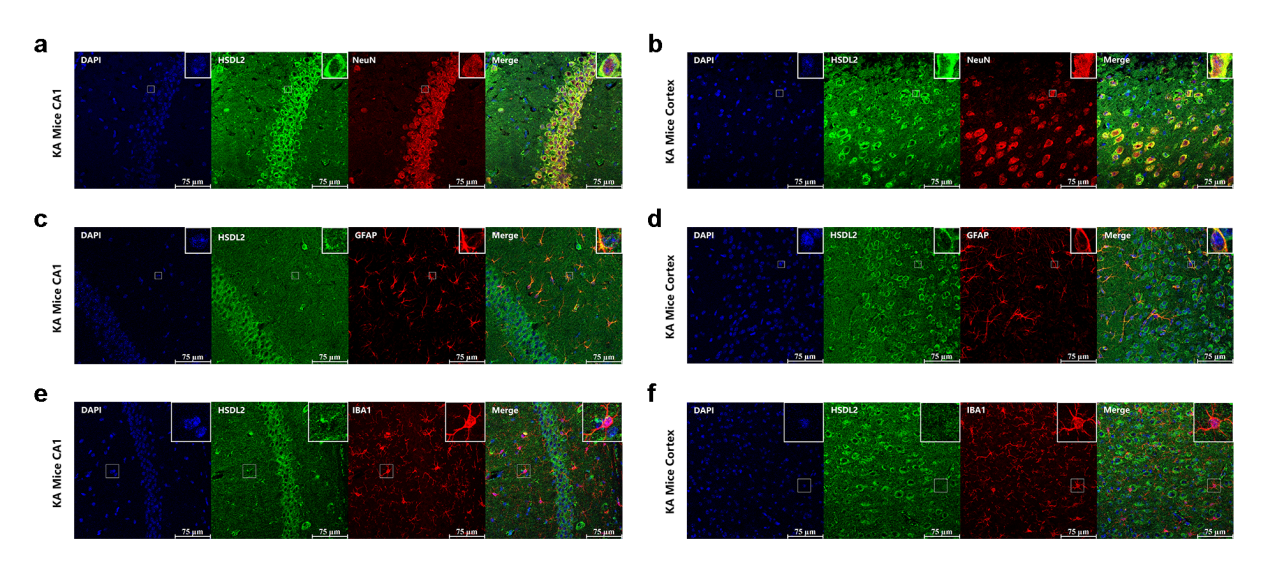


**Fig. S2:** **Distribution and localization of HSDL2 in KA mice brain tissues.**

**(a-b)** Representative immunofluorescence images of HSDL2, DAPI and NeuN in the hippocampus and cortex of KA mice.

**(c-d)** Representative immunofluorescence images of HSDL2, DAPI and GFAP in the hippocampus and cortex of KA mice.

**(e-f)** Representative immunofluorescence images of HSDL2, DAPI and IBA1 in the hippocampus and cortex of KA mice.


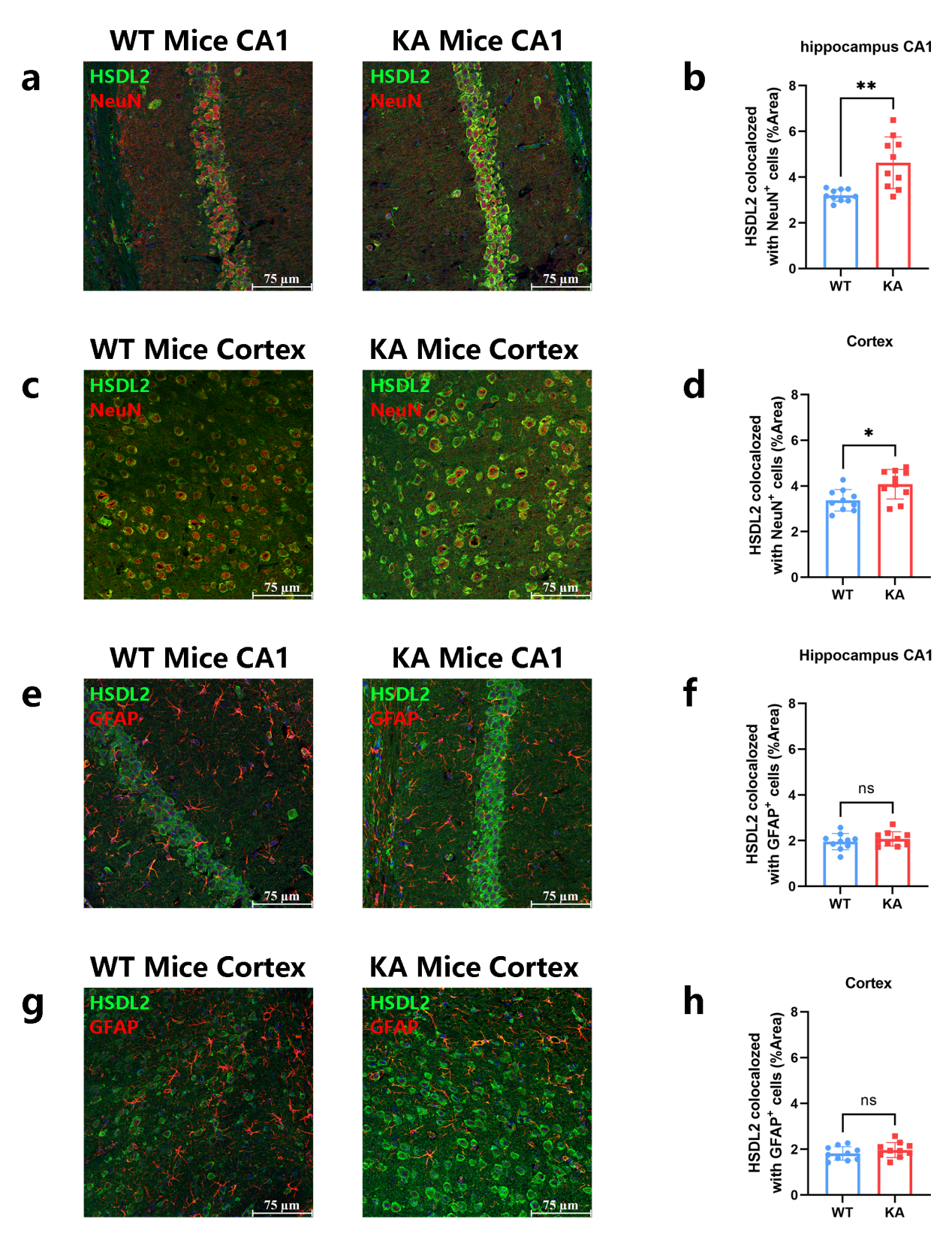


**Fig.S3:** **Quantitative Fluorescence Analysis of HSDL2 in Brain Sections of KA Mice and control Mice.**

**(a-b)** Immunofluorescence analysis in brain sections of KA mice revealed increased co-localization of HSDL2 with neurons in the hippocampal region compared to the control mice (n = 3/group, **P < 0.01).

**(c-d)** Immunofluorescence analysis in brain sections of KA rats revealed increased co-localization of HSDL2 with neurons in the cortical region compared to the control mice (n = 3/group, *P < 0.05).

**(e-f)** The co-localization of HSDL2 with astrocytes in the hippocampal region showed no statistically significant difference compared to the control mice (n = 3/group, ns P>0.05).

**(g-h)** The co-localization of HSDL2 with astrocytes in the cortical region showed no statistically significant difference compared to the control mice= 3/group, ns P>0.05).

**
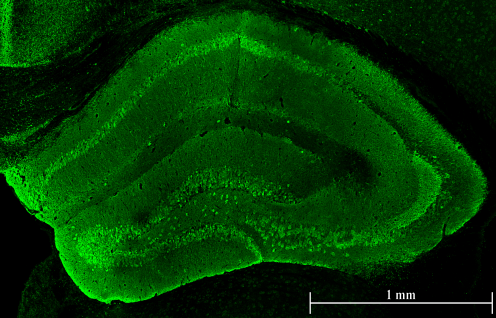
**

**Fig.S4: Immunofluorescence staining of enhanced green fluorescent protein (eGFP) in the hippocampus following transfection with AAV-eGFP-adHSDL2 (overexpression) or AAV-eGFP-siHSDL2 (knockdown).**

**
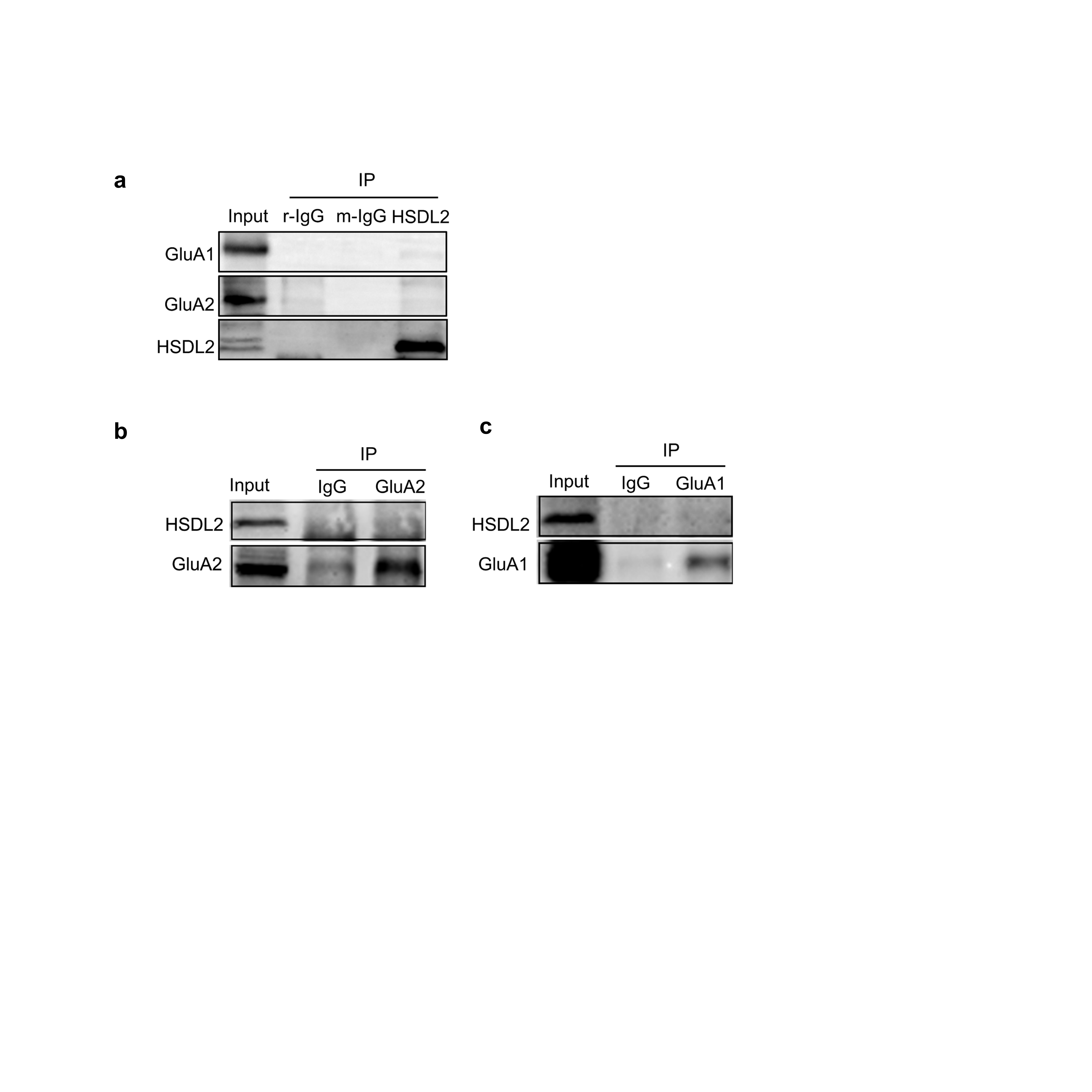
**

**Fig.S5: Co-immunoprecipitation (Co-IP) assays confirming physical interactions between HSDL2 and GluA1and GluA2 subunits.**

1. Hippocampal lysates from wild-type mice were precipitated with anti-HSDL2 magnetic beads, and precipitates were subsequently immunoblotted with antibodies to detect GluA1 and GluA2. IgG immunoprecipitate was used as a negative control.
   **(b-c)** Reverse validation of the interaction of GluA1 and GluA2 with HSDL2 in lysates from the hippocampal lysates from wild-typel mice by Co-IP assay.

**Supplementary Table**

**Table S1. Antibody details.**

| **Antibody** | **Company** | **Catalog Number** | **Source** | **Applications** |  |
| --- | --- | --- | --- | --- | --- |
| HSDL2 | FineTest  (Wuhan, China) | FNab04034 | Rabbit | WB (1:1000)  IF (1:50) |  |
| GAPDH | Proteintech  (Wuhan, China) | 60004-1-Ig | Mouse | WB (1:5000) |  |
| β-tubulin | Proteintech | 80713-1-RR | Rabbit | WB (1:5000) |  |
| Na-K-ATP | Proteintech | 14418-1-AP | Rabbit | WB (1:5000) |  |
| GluN2B | Proteintech | 21920-1-AP | Rabbit | WB (1:1000) |  |
| GluN2A | Proteintech | 28525-1-AP | Rabbit | WB (1:1000) |  |
| GluN1 | Proteintech | 27676-1-AP | Rabbit | WB (1:1000) |  |
| GluA1 | Proteintech | 67642-1-Ig | Mouse | WB (1:1000) |  |
| GluA2 | Proteintech | 11994-1-AP | Rabbit | WB (1:1000) |  |
| PSD95 | ThermoFisher | MA1-046 | Mouse | IF (1:500) |  |
| PSD95 | Zen-bio  (Chengdu, China) | R381001 | Rabbit | WB (1:500) |  |
| Phospho-(Ser/Thr) Phe Antibody | CST  (USA) | 9631S | Rabbit | WB (1:1000) |  |
| GFAP | Proteintech | 60190-1-Ig | Mouse | IF (1:200) |  |
| NeuN | Proteintech | 66836-1-Ig | Mouse | IF (1:200) |  |
| HRP-conjugated Goat Anti-Rabbit IgG | Proteintech | SA00001-2 | Goat | WB (1:8000) |  |
| Dylight 594, Goat Anti-Mouse IgG | Abbkine  (California, USA) | A23410 | Goat | IF (1:200) |  |
| Dylight 488, Goat Anti-Rabbit IgG | Abbkine | A23220 | Goat | IF (1:200) |  |

**Table S2. Sequences of qPCR primers.**

| primers | Sequence（5’ to 3’） |
| --- | --- |
| Mouse HSDL2 (F) | GCTATGGATATGCTGGGAGGATC |
| Mouse HSDL2 (R) | AACTGCATCTGGGTGTTCATCTA |
| Mouse GAPDH (F) | CACCCACTCCTCCACCTTTGAC |
| Mouse GAPDH (R) | GTCCACCACCCTGTTGCTGTAG |

**Table S3. Clinical characteristics of TLE patients and TBI patients.**

| Cases | Gender  (M/F) | Age  (years) | Course  (years) | AEDs before surgery | Resection tissue | Neuropathological  diagnosis |
| --- | --- | --- | --- | --- | --- | --- |
| TLE 1 | M | 22 | 18 | VPA, CBZ, PHT | TN, L | NL, G |
| TLE 2 | F | 21 | 8 | VPA, CBZ, TPM | TN, L | G |
| TLE 3 | F | 26 | 7 | VPA, CBZ, TPM | TN, L | G |
| TLE 4 | M | 42 | 16 | PB, VPA, CBZ, OXC | TN, R | NL, G |
| TLE 5 | F | 21 | 6 | VPA, CBZ, TPM, RHT | TN, R | NL |
| TLE 6 | F | 17 | 9 | VPA, CBZ, PB | TN, R | NL, G |
| TLE 7 | M | 12 | 3 | VPA, CBZ, PB | TN, L | NL |
| TLE 8 | F | 29 | 11 | VPHT, LGT, TPM, CBZ | TN, L | NL, G |
| TLE 9 | M | 18 | 4 | PHT CBZ, LEV | TN, R | G |
| TLE 10 | F | 22 | 13 | VPA, CBZ, TPM | TN, R | NL, G |
| TLE 11 | M | 18 | 16 | VPA, LEV, TPM | TN, R | NL, G |
| TLE 12 | F | 14 | 9 | VPA, CBZ, PB | TN, R | NL, G |
| TBI 1 | M | 35 | 0 | None | TN, L | N |
| TBI 2 | M | 25 | 0 | None | TN, R | N |
| TBI 3 | F | 31 | 0 | None | TN, R | N |
| TBI 4 | M | 11 | 0 | None | TN, L | N |
| TBI 5 | M | 22 | 0 | None | TN, R | N |
| TBI 6 | M | 44 | 0 | None | TN, L | N |
| TBI 7 | F | 17 | 0 | None | TN, R | N |
| TBI 8 | F | 2 | 0 | None | TN, R | N |
| TBI 9 | M | 34 | 0 | None | TN, L | N |
| TBI 10 | F | 54 | 0 | None | TN, L | N |

AEDs, antiepileptic drugs; CBZ, carbamazepine; CZP, clonazepam; F, female; G, gliosis; L, left; LTG, lamotrigine; M, male; N, relative normal; NL, neuron loss; OXC, oxcarbazepine; PB, phenobarbital; PHT, phenytoin; R, right; TBI, traumatic brain injury; TLE, temporal lobe epilepsy; TN, temporal neocortex; TPM, topiramate; VPA, valproate.
